# Application of PZT Technology and Clustering Algorithm for Debonding Detection of Steel-UHPC Composite Slabs

**DOI:** 10.3390/s18092953

**Published:** 2018-09-05

**Authors:** Banfu Yan, Qiqi Zou, You Dong, Xudong Shao

**Affiliations:** 1 College of Civil Engineering, Hunan University, Changsha 410006, China; yanbanfu@hnu.edu.cn (B.Y.); shaoxd@hnu.edu.cn (X.S.); 2 Department of Ocean Science and Engineering, Southern University of Science and Technology, Shenzhen 518055, China; 11849508@mail.sustc.edu.cn; 3 Department of Civil and Environmental Engineering, The Hong Kong Polytechnic University, Hong Kong, China

**Keywords:** steel-UHPC, composite slab, debonding detection, PZT technology, clustering algorithm

## Abstract

A lightweight composite bridge deck system composed of steel orthotropic deck stiffened with thin Ultra-High Performance Concrete (UHPC) layer has been proposed to eliminate fatigue cracks in orthotropic steel decks. The debonding between steel deck and UHPC layer may be introduced during construction and operation phases, which could cause adverse consequences, such as crack-induced water invasion and distinct reduction of the shear resistance. The piezoelectric lead zirconate titanate (PZT)-based technologies are used to detect interfacial debonding defects between the steel deck and the UHPC layer. Both impedance analysis and wave propagation method are employed to extract debonding features of the steel-UHPC composite slab with debonding defect in different sizes and thicknesses. Experimental tests are performed on two steel-UHPC composite slabs and a conventional steel-concrete composite deck. Additionally, an improved Particle Swarm Optimization (PSO)-k-means clustering algorithm is adopted to obtain debonding patterns based on the feature data set. The laboratory tests demonstrate that the proposed approach provides an effective way to detect interfacial debonding of steel-UHPC composite deck.

## 1. Introduction

The lightweight steel-Ultra-High-Performance Concrete (UHPC) composite bridge deck, composed of steel deck and a thin reinforced UHPC layer through stud shear connectors, is an effective way to eliminate bridge defects, such as preventing pavement cracking and reducing fatigue cracking of the orthotropic steel deck [1,2,3,4]. In practice, however, construction of this type of novel structure is very challenging due to several reasons. First, the thickness of the UHPC layer in the composite bridge is relatively thin, i.e., 35–50 mm, and the composite slab requires a high-temperature steam-curing treatment. Second, the steel-fiber volume ratio in the structure could be up to 3.5%, which may cause steel fiber clustering. Third, the high-temperature steam curing can cause debonding between steel and the UHPC layer. Finally, the performance of the steel-UHPC composite deck system may degrade due to the effects of vehicle overloading, thermal action, and fatigue load action during operation. The debonding between the steel deck and the UHPC layer may introduce risks, such as crack-induced water invasion and distinct reduction of the shear resistance of the bridge deck system. Thus, it is of vital importance to effectively detect the interface debonding of the steel-UHPC deck system.

The piezoelectric ceramic patches (PZT) sensor, made of piezoelectric ceramics with both positive and negative piezoelectric effects, can be used simultaneously as an actuator and a sensor. Due to its lightweight characteristics, the PZT sensor can be installed on the surface of existing structures or embedded into newly-built structures for damage detection. It has been demonstrated to be particularly useful within civil engineering due to its unique features, such as active sensing, high-sensitivity, low cost, quick response, among others. In recent years, the PZT-based approach has been broadly recognized as one of the most promising, non-destructive evaluation methods for local damage identification [5,6].

The PZT-based damage identification methods can be classified into two categories: impedance-based method and vibration-characteristic-based wave propagation (WP) method. The impedance method aims to assess the status of a structure through the mechanic-electric coupling between the piezoelectric material and the host structure. The piezoelectric coupling properties of the piezoelectric materials can combine the structural mechanical impedance with the electric impedance of the piezoelectric materials. Thus, structural damage and condition can be realized by monitoring the change of electric impedance associated with the PZT patch installed on the surface or inside a structure. Over the past decades, many studies have been conducted on the application of the PZT impedance method for structural damage detection (e.g., [7,8,9,10,11,12,13,14]). For instance, Sun et al. [7] proposed a frequency domain impedance-signature-based technique for health monitoring of an assembled truss structure and used PZT as integrated sensor-actuators; Liang et al. [8] developed a coupled electro-mechanical analysis of piezoelectric ceramic actuators integrated in mechanical systems to determine the power consumption and energy transfer in electro-mechanical systems; Yang et al. [9] conducted an experimental study on local damage detection of beams and plates using PZT and demonstrated that both the location and extent of damage can be simultaneously identified; Xu et al. [12] investigated the structural crack damage using the impedance spectra of the PZT sensor, and presented a scalar damage metric based on the impedance spectra of the PZT sensor; and Sevillano et al. [13] proposed an innovative hierarchical clustering analysis to obtain a set of clusters based on damage patterns obtained from the PZT sensor.

With respect to the vibration -based WP method, the piezoelectric actuators generate stress wave under external excitation, which can be received by the piezoelectric sensors. Vibration features extracted from the acquired stress wave, such as changes in signal strength, arrival time, and transfer function before and after the introduction of damage, can be used for detection of structural damage. For instance, Wang et al. [15] used an active diagnostic technique for identifying impact damage in composite plates. This technique used a built-in network of piezoelectric actuators and sensors to generate and receive propagating stress waves over a wide range of frequencies; Roh and Chang [16] developed a diagnostic technique to detect the location and size of anomalies in isotropic plates; Wang [17] developed an active diagnostic system to detect embedded damage in fiber-reinforced composites and steel-reinforced concrete; and Song et al. [18] used piezoceramic transducers for damage detection of a reinforced concrete bridge bent-cap. During the experimental test, one embedded piezoceramic patch was used as an actuator to generate high frequency waves, and the other piezoceramic patch worked as a sensor to detect propagating waves; Lim et al. [19] performed experimental studies to investigate the application of the wave propagation method for concrete curing and monitoring of strength development; Lu et al. [20] investigated the propagation of ultrasonic waves in rebar-reinforced concrete beams for damage detection. An experimental test demonstrated that the surface-attached PZT disks were able to detect the change in material properties due to the existence of cracking; Xu et al. [21] proposed an active interface condition monitoring approach for concrete-filled steel tube (CFST) using functional smart aggregates as an actuator and PZT patches bonded on the surface of the steel tube as sensors.

Though researchers have conducted many studies on damage detection of different types of civil structures using piezoelectric impedance technology, wave propagation method, and clustering algorithm, there are limited studies on integration of the above proposed technologies for nondestructive evaluation. The lightweight steel-UHPC composite bridge deck system, as an effective and novel structural form to prevent bridge pavement cracking and reduce fatigue cracking of the orthotropic steel deck, faces the challenge of identifying the interfacial debonding condition between the steel deck and UHPC overlay. To the best knowledge of the authors, detection of the interfacial debonding of the steel-UHPC composite slab using PZT methods has not been studied.

In this study, both impedance analysis and wave propagation method are employed to extract the debonding features of steel-UHPC composite slab with different preset debonding defects. Additionally, an improved PSO-k-means clustering algorithm is adopted to obtain the clustering centers of the feature data set, and Mahalanobis distance is then used to distinguish the debonding degree of the deck system. The proposed methodology is validated through experimental tests on two steel-UHPC composite slabs and a conventional steel-concrete composite slab with different artificial debonding defects.

## 2. Experimental Work

### 2.1. Specimen and Sensor Installation

**Figure 1:**
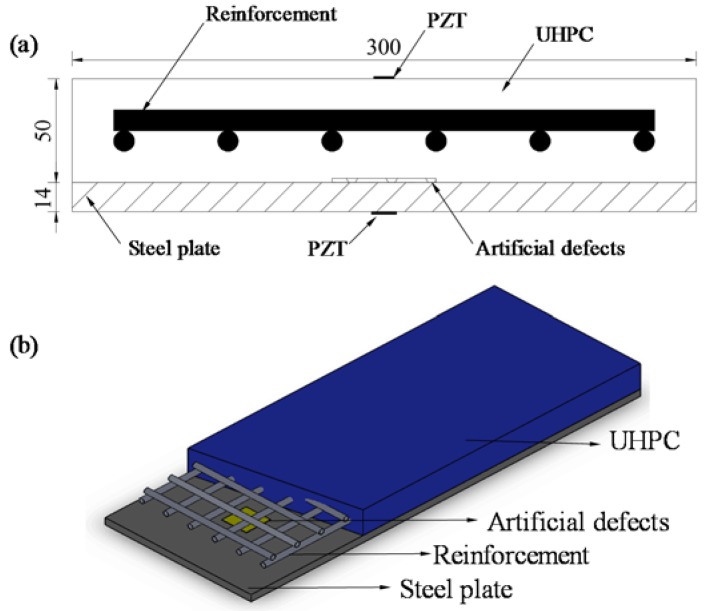
(**a**) Cross section and (**b**) setup of the specimen.

Three specimens termed as A-type (steel-UHPC composite slab with different preset debonding sizes), B-type (steel-UHPC composite slab), and C-type (conventional steel-concrete composite slab with different debonding thicknesses) are dimensioned as 800 mm (length) × 300 mm (width) × 64 mm (height), respectively. As shown in Figure 1, the specimen consists of a bottom steel plate with a thickness of 14 mm and a top thin reinforced UHPC layer with a thickness of 50 mm. The bottom steel plate is associated with a yield strength of 345 MPa. The UHPC is reinforced by HRB400 rebar with a diameter of 10 mm and spaced at 50 mm in both longitudinal and transverse directions. Before casting the 50 mm-thick UHPC overlay, the styrofoam sheets with different sizes and thicknesses are embedded in steel-UHPC interface as artificial defects to simulate the interfacial debonding.

**Figure 2:**
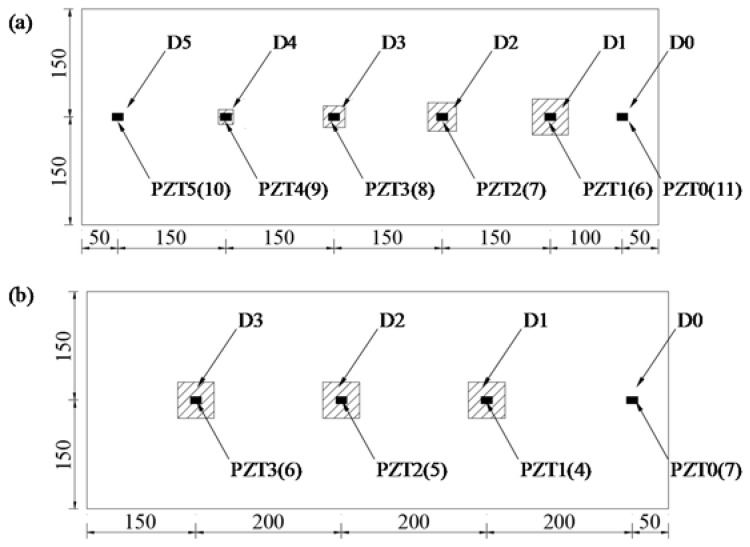
Setup of debonding areas and PZT patches for (**a**) A-type specimen and (**b**) B-type specimen (unit: mm).

As shown in Figure 2a and Table 1, the A-type specimen is configured with 5 pieces of styrofoam sheet as debonding defects. The sheets, ranging from 10 mm × 10 mm (D5) to 50 mm × 50 mm (D1) in dimension and 2 mm in thickness, are uniformly distributed along the length of specimen with a distance of 150 mm. Five pairs of PZT patches located within the debonding areas (D1–D5) and a pair of PZT patches outside the debonding areas (intact area: D0) are bonded symmetrically on the bottom side of the steel plate (debonding area: PZT1–PZT5; intact area: PZT0) and top surface of the UHPC layer (debonding area: PZT6–PZT10; intact area: PZT11).

As indicated in Figure 2b and Table 1, the B-type steel-UHPC plate with identical dimension and reinforcement of A-type specimen is embedded with 3 pieces of 50 mm × 50 mm styrofoam sheets. The thicknesses of the sheets are set to be 1 mm, 2 mm, and 3 mm, respectively. The number in brackets (Figure 2b) aims to distinguish the PZT patches attached on the top surface of UHPC. Moreover, in order to investigate the difference of PZT sensor signatures between the steel-UHPC and steel-NSC composite structures, as well as the effectiveness of the identification methods (e.g., PZT impedance and WP technique), a C-type specimen is constructed with identical dimension, defect setup and sensor placement of the B-type specimen, except that the overlay is composed of normal strength concrete.

### 2.2. Instrumental Setup

#### 2.2.1. Impedance Method

**Figure 3:**
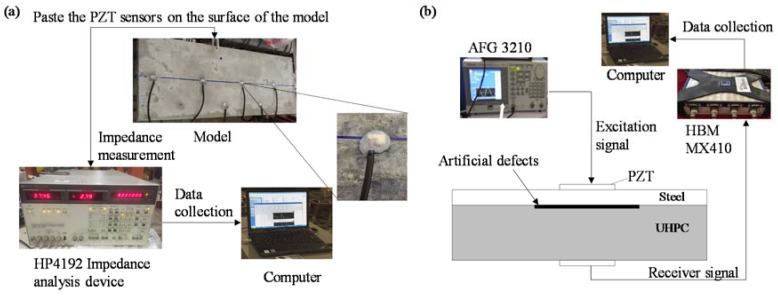
Setup of the testing system of (**a**) impedance method and (**b**) wave propagation method.

The instrumental setup is shown in Figure 3a. The impedance analyzer is employed to measure the admittance signatures of the PZT sensor on the surface of the steel plate. The PZT sensor is excited by alternating voltage with constant amplitude at preset frequency bands from the impedance analyzer. The impedance signature of the electromechanical coupling system composed of PZT patch, steel plate, and UHPC overlay is transmitted and stored by the laptop via GPIB connection. Features sensitive to debonding degree are extracted from the electromechanical coupling impedance-frequency curves of the PZT sensors at different damage locations. Then, the extracted features are used to identify the debonding defects in the steel-UHPC composite structure. Table 2 shows the main properties of the high-sensitivity PZT sensor with a dimension of 15 mm (length) × 10 mm (width) × 0.3 mm (thickness).

#### 2.2.2. Wave Propagation Method

The wave propagation measurement system, as indicated in Figure 3b, consists of PZT sensors, arbitrary waveform/function generator (Tektronix AFG3210), MX410 dynamic signal amplifier (HBM), testing specimen, and laptop. A sweep sinusoidal signal with a preset frequency bands generated by AFG3210 waveform/function generator is imposed on the PZT transducer bonded on the bottom surface of the steel plate. The stress wave propagates through the steel-UHPC composite structure with preset debonding defects and is acquired individually by the PZT sensor bonded on the top surface of UHPC overlay through a high-speed data acquisition system MX410 dynamic signal amplifier. The debonding defects are then identified through analyzing the variation of vibration parameters, such as signature amplitude, frequency spectral, energy distribution, among others.

### 2.3. Testing Scenarios

The PZT impedance technique is employed to measure the high frequency local impedance, which is sensitive to local damage of the structure. Table 3 shows the testing scenarios of the PZT impedance-based identification technique. Three-type specimens are tested under different scenarios, such as frequency bands of the impedance analyzer, location of PZT sensor, material type of overlay (e.g., UHPC and NSC), and thickness and size of the preset debonding defect. The sensitive features are then extracted from the impedance-frequency curves of the PZT sensors.

With respect to the wave propagation method, the propagation of stress wave in the structure is affected by three main factors, i.e., excitation voltage amplitude, excitation frequency, and propagation media (e.g., UHPC and NSC). Effects of these three factors on damage identification accuracy are investigated in the following section. The testing scenarios for the wave propagation method are shown in Table 4. The B-type specimen is employed to attain the appropriate excitation voltage amplitude and excitation frequency for accurate identification. The three-type testing specimens—A, B, and C—are tested to investigate the effects of material type, thickness, and size of debonding defects on identification accuracy under the excitation frequencies of 10 kHz (or 15 kHz) and the excitation voltage amplitude of 5 V.

## 3. Experimental Results

### 3.1. Impedance Method

#### 3.1.1. Excitation Frequency Bands

**Figure 4:**
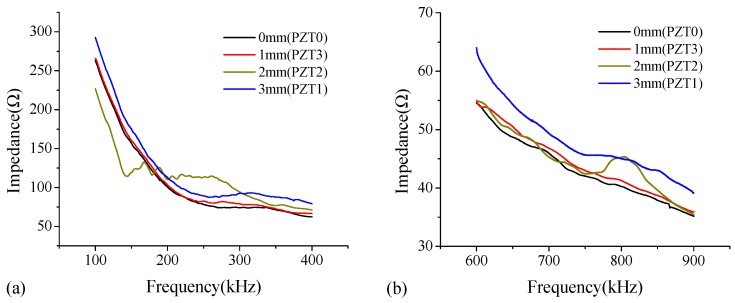

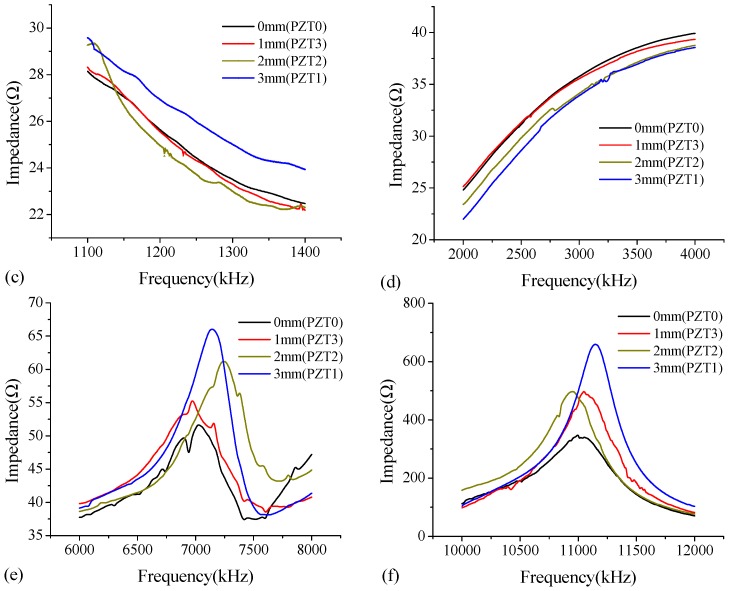
Impedance curve of PZT sensors bonded on the steel plate surface of the B-type specimen under excitation frequency bands of (**a**) 100–400 kHz; (**b**) 600–900 kHz; (**c**) 1100–1400 kHz; (**d**) 2000–4000 kHz; (**e**) 6000–8000 kHz; and (**f**) 10,000–12,000 kHz.

In the study, the thickness modes (Yang et al. [22], Park et al. [23]) of PZT patches are employed for debonding detection of the steel-UHPC composite slab. In order to select suitable excitation frequency bands, the PZT patches on the test specimen are scanned over a wide frequency range of 100–12,000 kHz. Among the roughly selected frequencies, the frequencies with obvious features, e.g., wide fluctuation, dominant peaks and troughs, and trend change, are scanned at a small interval. This procedure is iteratively applied until a proper frequency range is obtained. As shown in Figure 4, among the six frequency bands, the significant fluctuations and shifts of dominant peaks in impedance curves are observed at two frequency bands of 6000–8000 kHz (Figure 4e) and 10,000–12,000 kHz (Figure 4f). Note that these two frequency bands of 100–400 kHz (Figure 4a) and 600–900 kHz (Figure 4b) also give variations in the resonant frequency shifts of the signals and the frequency peaks; however, for the frequency ranges of 6000–8000 kHz (Figure 4e) and 10,000–12,000 kHz (Figure 4f), the obvious features such as the dominant frequency peaks and frequency fluctuation appear to be much more significant than those at the other frequency bands.

#### 3.1.2. Sensitivity of PZT at Different Locations

**Figure 5:**
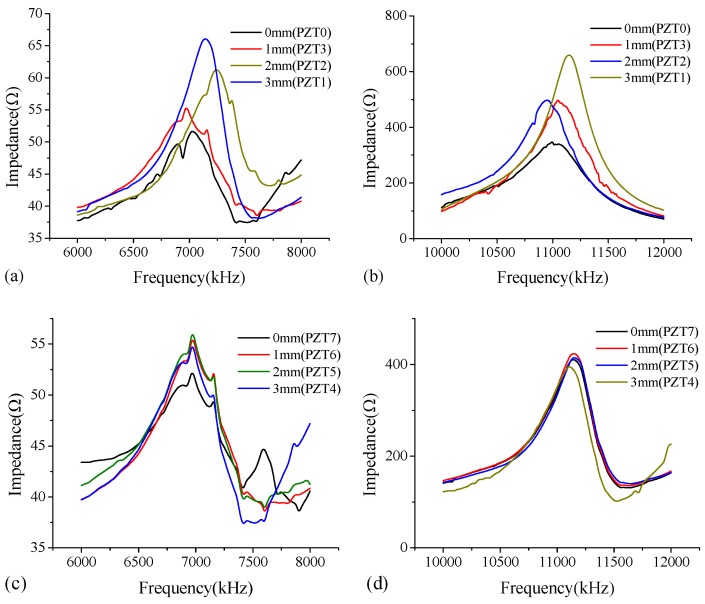
Impedance curves of PZT sensors bonded on the steel plate surface of the B-type specimen under the excitation frequency bands of (**a**) 6000–8000 kHz; (**b**) 10,000–12,000 kHz; Impedance curve of PZT sensors bonded on the UHPC surface of the B-type specimen under the excitation frequency bands of (**c**) 6000–8000 kHz; and (**d**) 10,000–12,000 kHz.

Figure 5 shows the impedance curves of the PZT sensors bonded on the steel plate surface and UHPC surface of the B-type specimen under the appropriate excitation frequency bands of 6000–8000 kHz and 10,000–12,000 kHz. It can be concluded that the differences of the impedance curves between the PZT patches bonded on the steel plate side (see Figure 5a,b) and those on the UHPC side (see Figure 5c,d) are distinct, and the significant fluctuation and shift of dominant peaks of the curves for PZT sensors bonded on the steel plate side are observed. This attributes to the fact that the impedance-based method is a type of local measurement technique. The thickness of the steel plate is far smaller than that of the UHPC overlay, and thus the impedance signatures at the steel plate side exhibit more appreciable sensitivity in the resonant frequencies and peak values than those at the UHPC side.

#### 3.1.3. Steel-UHPC vs. Steel-NSC

**Figure 6:**
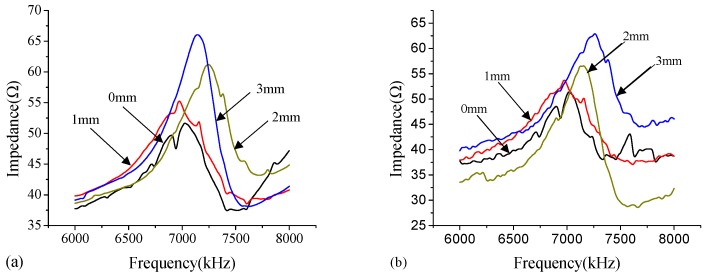
Impedance curve of the PZT sensors bonded on the steel plate surface of: (**a**) B-type specimen (Steel-UHPC) and (**b**) C-type specimen (Steel-NSC) under the excitation frequency band of 6000–8000 kHz.

The electrical impedance signature of the PZT patch, which is coupled with the mechanical impedance of host structure, could be affected by physical changes of structural mass, stiffness, and damping. For B-(steel-UHPC) and C-type (steel-NSC) specimens, the material type (i.e., UHPC or NSC) and the debonding defects could affect the impedance signature. Note that the surface-bonded PZT patches at the steel plate side of the B- and C-type specimens are close to the artificial debonding defects. The debonding defects rather than the material type may dominate the change of impedance signature. Figure 6 shows the impedance curves of the PZT sensors bonded on the steel plate surface of the B-type specimen (Steel-UHPC, Figure 6a) and C-type specimen (Steel-NSC, Figure 6b) under the excitation frequency band of 6000–8000 kHz. It can be concluded that the significant fluctuation and dominant peaks of the impedance curves are observed both in B- and C-type specimens. In addition, there is no distinct difference between the impedance curves for B-type specimen and those for C-type specimen with different debonding defect thicknesses of 0 mm, 1 mm, 2 mm, and 3 mm, respectively. This demonstrates that the effect of material type on the impedance curve is not dominant in this study.

Moreover, the EMI technique, especially when the PZT patch is bonded on metallic structure, is very sensitive to changes of boundary condition associated with the host structure [24]. Further studies are needed to assess the sensitivity of boundary condition within the EMI technique.

#### 3.1.4. Damage Index: Debonding Degree

It can be seen from the PZT impedance analysis that the debonding defect of the specimen with different sizes and thicknesses can be preliminarily identified through the variation of resonance frequency and impedance value of the PZT patch. However, this fails to quantitatively evaluate the debonding defect. The Root-mean-square deviation (RMSD) is then adopted as the damage index to identify the difference of signature between the intact and damaged state [25,26]. The effect of debonding defect in the specimen on impedance signature is the appearance of new peak and the fluctuation and shift of the existing peak in the impedance curve. In the study, RMSD of the PZT patches in different frequency ranges is employed as the damage index to quantify the debonding degree of the specimen. The damage index is defined as:

(1) $$\mathrm{RMSD}=\sqrt{\frac{\sum_{1}^{N}{\left(Z_{i}^{1}-Z_{i}^{0}\right)}^{2}}{\sum_{1}^{N}{\left(Z_{i}^{0}\right)}^{2}}}\times 100\%\;$$

where *N* is the frequency number of the impedance curves in the selected frequency range; $Z_{i}^{0}$ is the real part of impedance signature at the *i*th frequency data point under intact condition; and $Z_{i}^{1}$ is the real part of the impedance signature under the damaged condition. The used damage index represents the debonding degree of the composite structure. The index closes at 0, representing intact condition. The greater the index, the more severe the debonding defect.

**Figure 7:**
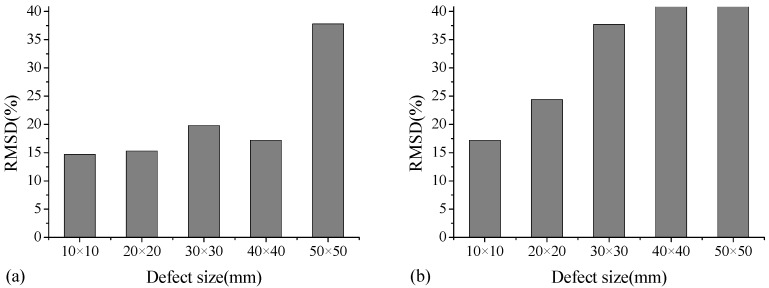
RMSD values of A-type specimen at the frequency ranges of (**a**) 6000–8000 kHz and (**b**) 10,000–12,000 kHz.

**Figure 8:**
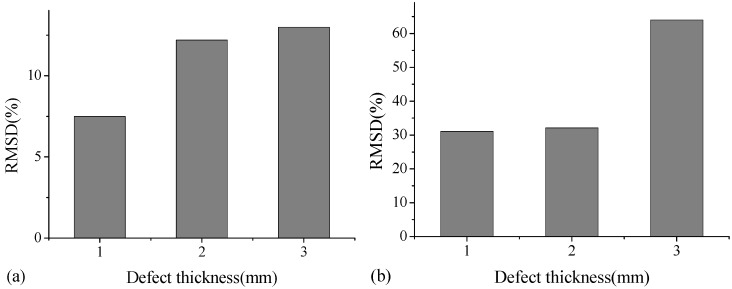
RMSD values of B-type specimen at the frequency ranges of (**a**) 6000–8000 kHz and (**b**) 10,000–12,000 kHz.

Figure 7 shows a typical RMSD value for A-type specimen with different debonding sizes at two frequency ranges of 6000–8000 kHz and 10,000–12,000 kHz. As indicated, the RMSD damage index in these two frequency ranges can reflect variations in debonding size. In the frequency range of 6000–8000 kHz for cases with defect size from 10 mm × 10 mm to 40 mm × 40 mm, the RMSD values appear to be in the range of 15–20%. With respect to the size of 50 mm × 50 mm, the RMSD increases to 38%. This indicates that in such frequency range, the damage index RMSD is not efficient to identify very minor debonding defects. Moreover, in the frequency range of 10,000–12,000 kHz, the RMSD value becomes relatively large at 38% when the debonding size approaches 30 mm × 30 mm. Figure 8 shows a typical RMSD value for B-type specimen with different debonding thicknesses at the frequency ranges of 6000–8000 kHz and 10,000–12,000 kHz. In these two frequency ranges, the RMSD damage index increases with the increase in debonding thickness. Therefore, although the RMSD damage indices exhibit little difference in different frequency ranges, the debonding defects in different sizes and thicknesses can be effectively identified.

### 3.2. Wave Propagation Method

#### 3.2.1. Amplitude of Excitation Signal

**Figure 9:**
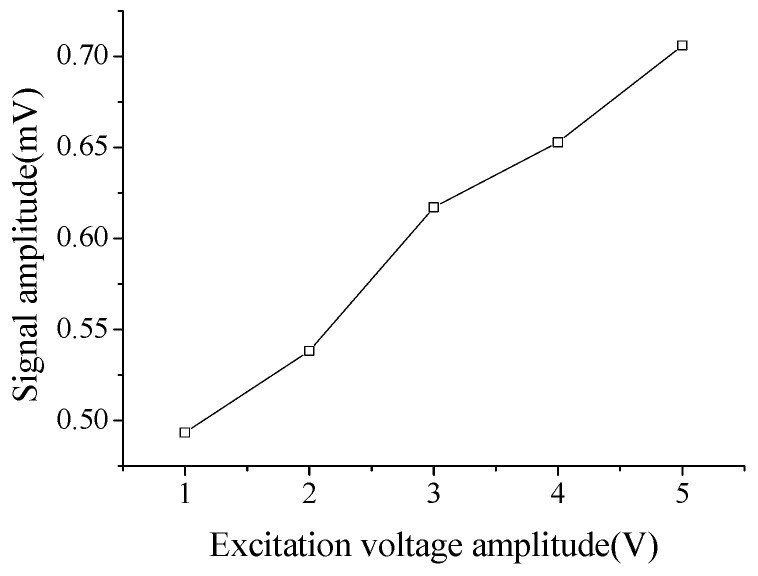
Relationship of signal amplitudes between the output and the input excitation voltage at the intact location of the B-type specimen.

It has been well recognized that the response of PZT sheet is proportional to the applied external force and electric field strength. The linearity of the PZT sensor is employed to investigate the linear relationship between the signal inputs and outputs. Figure 9 shows the relationship of the signal amplitudes between the output and input excitation voltage at the intact location for the B-type plate. It is observed that the linear relationship in this case is significant.

#### 3.2.2. Excitation Frequency

As presented by Sun et al. [27] and Lim et al. [28], the excitation frequency used in the WP technique for concrete structure is around 100 kHz. Note, that in this paper, the steel-UHPC composite slab is employed for investigation. The coupling effect between the PZT patches and steel-UHPC member and the effect of interface debonding defect is different from those for a pure concrete structure. Due to the existence of debonding defects, the higher excitation frequencies result in a rapid decay of the waveform amplitude. Thus, in this study, the ranges of input excitation frequencies are determined by achieving a balance between sensitivity and decay of waveform amplitude. 

**Figure 10:**
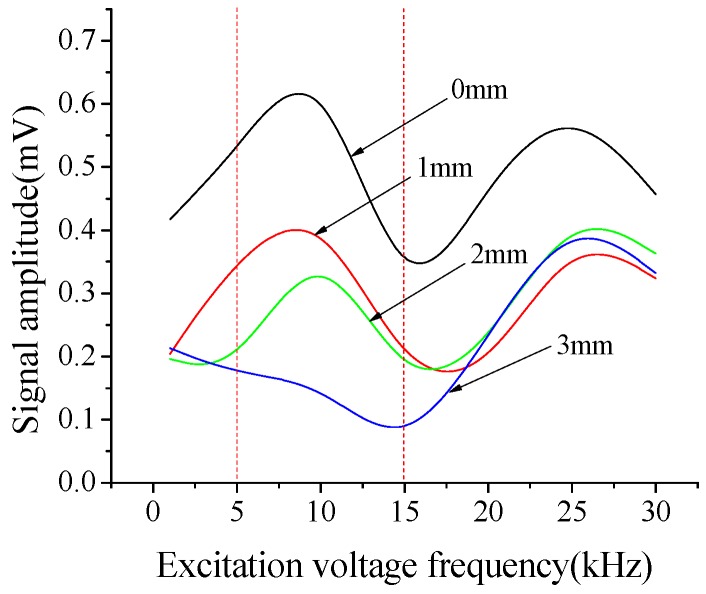
Relationship between the output signal amplitude and the excitation voltage frequency for the B-type specimen with different debonding thicknesses.

Figure 10 shows the relationship between the output signal amplitude and the excitation voltage frequency for B-type specimen with different debonding thicknesses. The candidate input excitation frequencies are set to be in the range of 1–30 kHz. As stated in Figure 10, when the excitation frequency is in the range of 5–15 kHz, the difference of the output voltage amplitude under different damage conditions is relatively large, which indicates that the energy dissipation of the excitation signal is more obvious when it travels through the debonding defects. In this study, the appropriate excitation frequency range is set to be 5–15kHz. The signals are recorded through a data acquisition system with a sampling frequency of 102.4 kHz.

#### 3.2.3. Steel-UHPC vs. Steel-NSC

**Figure 11:**
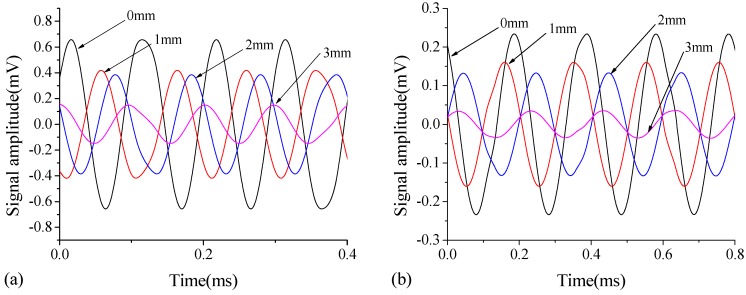
The receiver voltage of the output signals for (**a**) B-type steel-UHPC specimen and (**b**) C-type steel-NSC specimen with different debonding defects in thickness.

The sensitivity of the WP technique on identifying the interface debonding thickness of type-B steel-UHPC and C-type steel-NSC specimens from variations of receiver signal amplitudes is investigated in this section. The debonding defects of both B-type and C-type specimens have a size of 50 mm × 50 mm and thicknesses of 1 mm, 2 mm, and 3 mm. Figure 11 shows the receiver voltage of the output signals of these two specimens. It is observed that, the output voltage amplitudes of the PZT patches decrease significantly with increase of the thickness of debonding defects, compared with that at intact state for both B- and C-type specimens. Additionally, the signal amplitudes of the B-type specimen (Figure 11a) are larger than those of the C-type specimen (Figure 11b), which could be attributed to the fact that the B-type UHPC overlay with no coarse aggregate is denser than the normal strength concrete and has less energy dissipation.

#### 3.2.4. Output Voltage Amplitude

**Figure 12:**
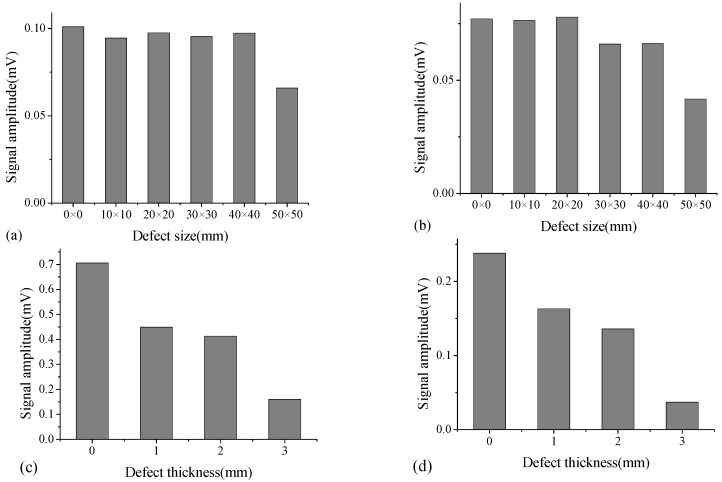
Output voltage amplitude for A-type specimen with different defect sizes under excitation voltage frequency of (**a**) 10 kHz; (**b**) 15 kHz, and those for B-type specimen with different defect thicknesses under excitation voltage frequency of (**c**) 10 kHz; and (**d**) 15 kHz.

The output voltage amplitude reflects the wave energy loss when a stress wave propagates through the interface. The size and thickness of the debonding defect are two key factors affecting the output voltage amplitude in the WP technique. Figure 12 shows the output voltage amplitudes for A-type specimen with different defect sizes (10 mm × 10 mm to 50 mm × 50 mm) under excitation voltage frequency of 10 kHz and 15 kHz, respectively. As indicated, amplitudes of the output voltage for cases with defect size from 10 mm × 10 mm to 40 mm × 40 mm appear to be identical to the intact case. With respect to the debonding size of 50 mm × 50 mm, the amplitude decreases dramatically. For B-type specimen, the defect sizes for all measurement points with different defect thicknesses are preset to be 50 mm × 50 mm. The amplitude of output voltage for B-type specimen under excitation frequencies of 10 kHz and 15 kHz is indicated in Figure 12c,d, respectively. The amplitudes decrease with the increase of the defect thicknesses from 1 mm to 3 mm. The minor changes in thickness of the defect affect the signatures significantly, which indicates the effectiveness of the WP technique in identifying the early age interfacial debonding defects with minor thicknesses. In addition, the excitation frequency can affect output voltage amplitude. The output voltage amplitudes exhibit differently; however, under the excitation frequency of 10 kHz and 15 kHz, the trend associated with the effect of the interface debonding size and thickness on output voltage amplitudes is consistent.

## 4. PSO-k-Means Clustering Based Debonding Detection

### 4.1. PSO-k-Means Clustering Algorithm

An improved PSO (Particle Swarm Optimization)-k-means clustering algorithm is developed to classify different debonding status of the steel-UHPC composite structure from the feature data set [29,30]. The method integrates the global searching ability of PSO method with the quick convergence characteristic of k-means clustering. The global search ability of the PSO algorithm is enhanced dynamically by changing the inertia weights of the particles. Particle zoning is determined by the nearest neighbor approach. The post convergence of the particles is accelerated through the quick searching of the *k*-means clustering approach. The fitness variance threshold and maximum number of iterations are used to determine the iterative execution. The ideal clusters are obtained from the global searching of optimal clusters in the particles. The number of clusters *k* = 4 and the number of particles *n* = 10 are taken. The particles are firstly initialized by randomly assigning a data sample as the initial cluster. The procedure is repeated *n* times. Cluster zoning is determined by the nearest-neighbor method. Given a data sample set $X_{i}$, if:

(2) $$\left\|X_{i}-C_{j}\right\|=\min\left\|X_{i}-C_{i}\right\|,(i=1,2\ldots ,k)\;$$

Then, $X_{i}$ belongs to cluster *j*. The parameter $C_{j}$ is the *j*th cluster. Given a particle, the fitness $F_{i}$ is the sum of the distance between the sample in the cluster and the clustering center, i.e.,

(3) $$F_{i}={\sum_{i=1}^{L}\sum_{j=1}^{k}\left\|X_{i}-C_{j}\right\|}^{2}\;$$

where *L* is the number of the clustering samples. After the initial clustering, the speed and location of particles are updated according to the learning factor and inertia weight coefficients. With the initial speed of zero, we have:

(4) $$V_{i}^{t+1}=\omega V_{i}^{t}+c_{1}r_{1}(P_{i}^{t}-X_{i}^{t})+c_{2}r_{2}(P_{g}^{t}-X_{i}^{t})\;$$

(5) $$X_{i}^{t+1}=X_{i}^{t}+V_{i}^{t}\;$$

where $V_{i}^{t}$ is the speed of particles; $X_{i}^{t}$ is the location of particles (*i* = 1, 2, …, *N*, in which *N* is the dimension of space); $P_{i}^{t}$ is the optimal position of particles; $P_{g}^{t}$ is the global optimal value; $r_{1}$ and $r_{2}$ are random values in [0, 1]; $c_{1}$ and $c_{2}$ are learning factors; and ω is the inertia weight, which is modified dynamically within the whole iterative process. In general, a larger inertia weight indicates a better global searching capability, while a smaller inertia weight indicates a better local searching capability. The linear iteration strategy is taken in this study with the preset maximum iteration number *itermax*. The inertia weight is updated as:

(6) $$\omega =\omega_{\max}-iter\times \frac{\omega_{\max}-\omega_{\min}}{itermax}\;$$

where *iter* is the current iteration and $\omega_{\max}=0.9$ and $\omega_{\min}=0.4$ are the maximum and minimum iterations, respectively. Through dynamically updating the inertia weight, the proposed algorithm can provide better global searching ability. The clusters are re-calculated after updating velocity and position. The fitness values of individual and global particles are also updated to the optimum.

### 4.2. Features and Samples Selection

As described previously, the impedance curves and output voltage amplitude of the debonding status with different thicknesses are obtained from mechanical impedance and WP methods. The curves and signatures contain characteristic features representing the damage status. The proposed clustering algorithm is then applied to obtain the clusters representing different damage statuses. The clustering center mathematically represents the shortest distance from each point in the cluster to the center. For a new test, the Mahalanobis distance is calculated for each testing data to the cluster center. The testing data is then classified into the clusters with the shortest distance, which can be used to identify the damage status and severity. The clustering centers comprehensively integrate different features obtained from impedance and WP methods.

As shown in Table 5, four features sensitive to the damage severities are extracted from the impedance analysis and wave propagation method, including two RMSD values of PZT patches in the frequency ranges of 6000–8000 kHz and 10,000–12,000 kHz and two output voltage amplitudes under the excitation frequencies of 10 kHz and 15 kHz. By repeating the tests, 50 samples are obtained from each status (intact status and three damage status with three different thickness values at the debonding interface), yielding 200 samples.

**Figure 13:**
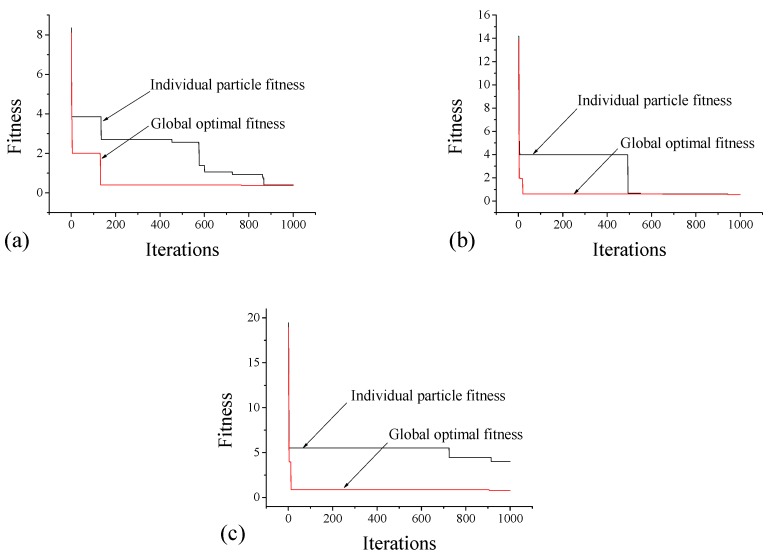
Fitness curves of individual representative particles and global best solutions for (**a**) case 7; (**b**) case 8; and (**c**) case 9 as indicated in Table 6.

The extracted feature characteristics and the number and quality of samples have a direct impact on identification accuracy. With the increase in the number of clustering samples and enhancement of sample qualities, the clustering centers are optimized continually, which results in accurate identification. As shown in Table 6, the feature vectors of each status for a total of 200 samples are randomly split into two separate sets for training and testing. The 30%, 50%, and 70% samples are selected for training and rest 70%, 50%, and 30% are considered to check the robustness of the clustering algorithm. The PSO-k-means clustering algorithm is employed to find the optimal clustering centers of the four statuses (intact status and three damage statuses with three different debonding thicknesses). Figure 13 shows variations of the optimal fitness functions for individual representative particles and the global best solution in different cases. Accordingly, the PSO-k-mean clustering algorithm has an outstanding global search ability. The optimal fitness curve tends to converge when the iteration number arrives at 160 for case 7, whereas in the cases of 8 and 9, the swarm is shown to take no more than 20 iterations to attain the best solution. 

### 4.3. Debonding Detection Using Mahalanobis Distance

**Figure 14:**
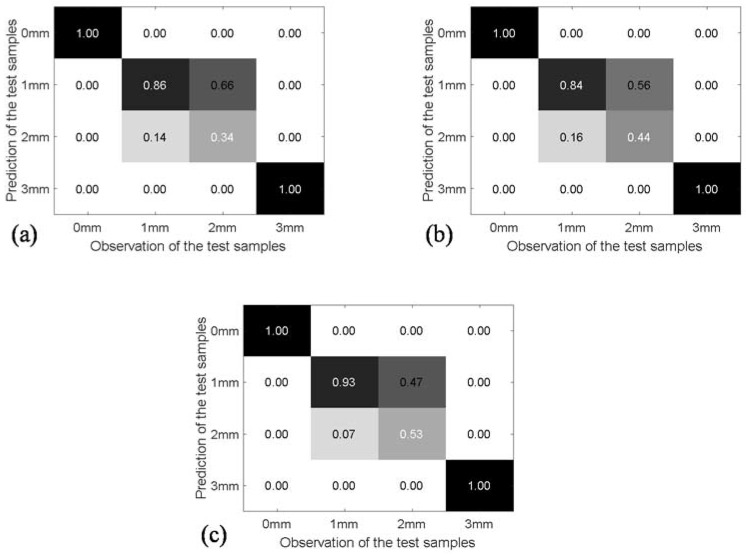
Confusion matrix for classification accuracies of PSO-k-means predictions on test samples of (**a**) case 7: 30% for training; (**b**) case 8: 50% for training; and (**c**) case 9: 70% for training.

As the optimal clustering center was attained for each damage status via the PSO-k-means algorithm, the Mahalanobis distance of each testing sample to the four clustering centers is calculated, and the testing sample is classified into the pattern (damage status) with the smallest Mahalanobis distance [31]. In the paper, the confusion matrix is employed to evaluate the overall identification accuracy of the PSO-k-means algorithm and Mahalanobis distance. This matrix contains information about actual and predicted classifications. For a *N*-class classification problem, the element of a confusion matrix *CM_i,j_* is equal to the ratio of the number of observations known to be in group *j* but predicted to be in group *i* to the total number of group *j*. Ideally, with 100% accuracy, the diagonal of confusion matrix is 1.0, which indicates that the predicted values fully match to their observation. Figure 14 shows the identification results of cases 7–9, determined in Table 6. It is observed that the debonding status with the thickness of 0 mm and 3 mm can be effectively detected. However, the feature characteristics of the testing samples with the debonding thickness of 1 mm and 2 mm are relatively close, which can result in a misclassification of the pattern. Moreover, with the increase of the training samples from 30% to 70%, the overall accurate classification rates for rest of the test samples increase from 80% to 86.5%.

Besides the split strategies for training and testing samples, the quality of clustering sample and number of features play an important role in pattern classification. In the study, to avoid the effect of sample quality on the identification accuracy rate, the identification processes are iterated for 100 times of randomly selecting training and testing sets. The average identification accuracy is employed as a significant indicator to evaluate the classifier. Table 7 shows the averaged identification accuracy rates under different features (e.g., different split strategies for training and the prediction test for the remaining samples). As indicated, when only the normalized impedance features RMSD in different frequency bands or the features (output voltage amplitude) from the wave propagation method are individually considered as the input attributes, the identification accuracy is obviously smaller than that when both of them are considered (e.g., cases 7–9). For instance, when features 1 and 2 are both considered, the average identification accuracy rates for case 1, 2 and 3 are 62.1%, 64.7%, and 67.8%, respectively. When only the features from wave propagation method are selected, the average identification accuracy rates reach 77.8–83.5%, which indicates that the wave propagation method can provide better classification result than the impedance-based PZT method. Moreover, as all four features are selected, the averaged identification rates attain 78.5–86.3%.

## 5. Concluding Remarks

This paper presents a detection method integrating PZT-based impedance analysis, wave propagation technique, PSO-k-means algorithm and Mahalanobis distance to identify the debonding defects of steel-UHPC composite deck. Both impedance analysis and wave propagation method are employed to extract debonding features of the steel-UHPC composite slab with debonding defect in different sizes and thicknesses. An improved PSO-k-means clustering algorithm is then used to obtain the clustering centers of the feature data set, and the Mahalanobis distance is finally used to distinguish the debonding degree of the samples. The proposed methodology is validated through experimental tests on two steel-UHPC composite slabs and a conventional steel-concrete composite slab with different debonding defects. 

The impedance test indicates that at two higher frequency ranges (i.e., 6000–8000 kHz, 10,000–12,000 kHz), the significant fluctuations and shifts of dominant peaks in impedance curves are observed. The RMSD is employed as an evaluation indicator of the debonding defect. Specially, as the debonding size of steel-UHPC interface reaches 50 mm × 50 mm, the RMSD damage index provides an effective tool to detect the debonding defect. Moreover, in such debonding size and appropriate frequency ranges, the RMSD damage index increases with the increase in debonding thickness.

The experimental tests indicate that the WP technique exhibits good performance in identifying defects of at least 50 mm × 50 mm in size and is capable of identifying early age interfacial debonding defects with minor thickness, e.g., 1.0 mm. In addition, the excitation frequency has an effect on the output voltage amplitude. The output voltage amplitudes exhibit distinct difference; however, under the excitation frequency of 10 kHz and 15 kHz, the trend of the effect associated with the interface debonding size and thickness on output voltage amplitudes is consistent.

The training and testing samples with four features extracted from impedance analysis and wave propagation method are considered for further PSO-k-means clustering analysis. The confusion matrix is employed to evaluate the overall identification accuracies of the PSO-k-means algorithm and Mahalanobis distance. It is observed that the debonding status with thickness of 0 mm and 3 mm can be effectively classified. However, the feature characteristics of the testing samples with debonding thickness of 1 mm and 2 mm are relatively close, which could result in misclassification. In addition, the wave propagation method can provide better classification results than the impedance-based PZT method. The averaged identification rates attain 86.3% when all four features are considered.

## Figures and Tables

**Table 1:** Details of PZT patches and specimen.

Specimen Labels	Size of Structures (Length × Width × Height)	Artificial Debonding Defects		Size of PZT Patch (Length × Width × Height)	PZT Patch Labels	
		Labels of Debonding Defect	Debonding Defect Size (Length × Width × Height)		Steel	UHPC (NSC)
A	800 mm × 300 mm × 64 mm	D0	Intact area	15 mm × 10 mm × 0.3 mm	PZT0	PZT11
		D1	50 mm × 50 mm × 2 mm		PZT1	PZT6
		D2	40 mm × 40 mm × 2 mm		PZT2	PZT7
		D3	30 mm × 30 mm × 2 mm		PZT3	PZT8
		D4	20 mm × 20 mm × 2 mm		PZT4	PZT9
		D5	10 mm × 10 mm × 2 mm		PZT5	PZT10
B (C)	800 mm × 300 mm × 64 mm	D0	Intact area	15 mm × 10 mm × 0.3 mm	PZT0	PZT7
		D1	50 mm × 50 mm × 1 mm		PZT1	PZT4
		D2	50 mm × 50 mm × 1 mm		PZT2	PZT5
		D3	50 mm × 50 mm × 1 mm		PZT3	PZT6

**Table 2:** Properties of the PZT sensor.

Properties	Values	Properties	Values
Piezoelectric strain factor (10–12 C/N)	450	Curie point (°C)	310
Relative dielectric constant	1800	Dielectric loss (%)	1.5
Electromechanical coupling factor	0.71	Density (g/cm^3^)	7.6
Mechanical quality factor	65		

**Table 3:** Testing scenarios associated with impedance method.

Testing Parameters	Testing Specimen	Measurement Frequency (kHz)
Measurement frequency bands	B	100–12,000
Location of testing points	B	6000–8000, 10,000–12,000
Material type (i.e., UHPC and NSC)	B, C	6000–8000
Thickness of debonding defects	B	6000–8000, 10,000–12,000
Size of debonding defects	A	6000–8000, 10,000–12,000

**Table 4:** Testing scenarios associated with wave propagation method.

Testing Parameters	Testing Specimen	Excitation Signal
Excitation voltage amplitude	B	1–5 V (10 kHz)
Excitation frequency	B	1–30 kHz (5 V)
Material type (i.e., UHPC and NSC)	B, C	10 kHz (5 V)
Thickness of debonding defects	B	10 kHz, 15 kHz (5 V)
Size of debonding defects	A	10 kHz, 15 kHz (5 V)

**Table 5:** Extracted features.

Item	Features	Source
1	RMSD (6000–8000 kHz)	Impedance method
2	RMSD (10,000–12,000 kHz)	Impedance method
3	Output voltage amplitude (10 kHz)	Wave Propagation method
4	Output voltage amplitude (15 kHz)	Wave Propagation method

**Table 6:** Split strategies for training and testing samples.

Case	Selected Features	Training Samples/(Percent)	Testing Samples
1	1, 2	60 (30%)	140
2	1, 2	100 (50%)	100
3	1, 2	140 (70%)	60
4	3, 4	60 (30%)	140
5	3, 4	100 (50%)	100
6	3, 4	140 (70%)	60
7	1, 2, 3, 4	60 (30%)	140
8	1, 2, 3, 4	100 (50%)	100
9	1, 2, 3, 4	140 (70%)	60

**Table 7:** Averaged rates of identification accuracy.

Cases	Selected Features	Percent of Training Samples		
		30%	50%	70%
1–3	1, 2	62.1%	64.7%	67.8%
4–6	3, 4	77.8%	81.1%	83.5%
7–9	1, 2, 3, 4	78.5%	81.8%	86.3%
